# Persistent morbidity in Clade IIb mpox patients: interim results of a long-term follow-up study, Belgium, June to November 2022

**DOI:** 10.2807/1560-7917.ES.2023.28.7.2300072

**Published:** 2023-02-16

**Authors:** Nicole Berens-Riha, Stefanie Bracke, Jojanneke Rutgers, Christophe Burm, Liesbeth Van Gestel, Matilde Hens, Chris Kenyon, Emmanuel Bottieau, Patrick Soentjens, Isabel Brosius, Marjan Van Esbroeck, Koen Vercauteren, Johan van Griensven, Christophe van Dijck, Laurens Liesenborghs, ITM monkeypox study group, Members of the ITM Monkeypox Study Group, Irith De Baetselier, Dorien Van den Bossche, Kevin K Ariën, Jasmine Coppens, Fien Vanroye, Kadrie Ramadan, Karin Van Looveren, Jolien Baeyens, Leo Heyndrickx, Hanne Rasson, Jacob Verschueren, Antonio Rezende, Leen Vandenhove, Bea Vuylsteke

**Affiliations:** 1 Institute of Tropical Medicine, Antwerp, Belgium; 2 University of Cape Town, Cape Town, South Africa; 3 The members of the study group are listed under Collaborators at the end of the article.; * These authors contributed equally to this work and share last authorship.

**Keywords:** mpox, monkeypox, follow-up, sequelae, medium-term follow-up, long-term follow-up, fatigue

## Abstract

While mpox was well characterised during the 2022 global Clade IIb outbreak, little is known about persistent morbidity. We present interim results of a prospective cohort study of 95 mpox patients assessed 3–20 weeks post-symptom onset. Two-thirds of participants had residual morbidity, including 25 with persistent anorectal and 18 with genital symptoms. Loss of physical fitness, new-onset/worsened fatigue and mental health problems were reported in 36, 19 and 11 patients, respectively. These findings require attention by healthcare providers.

In the 2022 global outbreak of mpox (formerly monkeypox), the majority of patients presented with nonspecific prodromal symptoms and localised skin and mucosal lesions, especially affecting the anal and genital regions [1,2]. Most infections were mild, with only 6% of cases requiring hospitalisation and only five recorded deaths out of 25,506 cases detected in the European Union region up to January 2023 [3]. Nevertheless, acute complications like bacterial superinfection, proctitis, penile oedema, urinary retention and paraphimosis were common [1,2,4,5] and severe manifestations were reported, especially among immunocompromised patients [6,7]. Moreover, the later consequences of the disease remain unknown. We, therefore, aimed to evaluate the medium- and long-term morbidity following the acute episode of mpox. In this interim report, we present the results of the medium-term follow-up (3–20 weeks after infection) of a cohort of mpox patients at a sexual health clinic in Antwerp, Belgium.

## Prospective follow-up of former mpox patients

Of over 300 mpox patients diagnosed at our clinic between May and October 2022, 169 mpox patients were interviewed during their first presentation and consented to be followed up. The follow-up study was designed to include the following visits: a first visit during the post-acute convalescent stage (intended 3–6 weeks post-symptom onset), and two later visits after 6 months and 1 year. Here, we present the interim results of the first visit only, as data collection for the subsequent visits is still ongoing.

Ninety-five (56.2%) of 169 participants attended the first visit (Table 1). Due to organisational and logistical reasons during the epidemic, only 61 (64.2%) of them attended within the foreseen time frame. The remaining 34 participants returned for their first visit after 7–20 weeks. For transparency, we report the results of these two groups of participants in this report as an ‘early’ and ‘late’ follow-up group, respectively.

**Table 1:** Population characteristics and severity assessment in mpox patients, Antwerp, Belgium, June–November 2022 (n = 169)

Characteristics	Time of assessment							
	At first visit/diagnosis				At follow-up			
	All mpox patients (n = 169)		Follow-up group (n = 95)		Follow-up group (3–20 weeks) (n = 95)		Early (3–6 weeks) (n = 61)	Late (7–20 weeks) (n = 34)
	n	%	n	%	n	%	n	n
Age in years, median (IQR)	39 (33–46)		39 (33–46)		39 (33–46)		40 (34–46)	39 (32.8–53)
Cis-MSM	169	100.0	95	100.0	95	100.0	61	34
Living with HIV^a^	57	33.7	28	29.5	28	29.5	16	12
HIV-negative on PrEP^b^	68/112	60.7	40/67	59.7	40/67	59.7	24/45	16/22
Assumed smallpox childhood vaccination (born before 1976)	36	21.3	22	23.2	22	23.2	12	10
Emergency room attendance	0	0	0	0	4	4.2	2	2
Complications^c^	17	10.1	7	10.3	57	60.0	38	19
WHO performance status^d^								
0	110	65.1	60	63.2	31	32.6	19	12
1	37	21.9	25	26.3	33	34.7	23	10
2	13	7.7	4	4.2	23	24.2	14	9
3	7	4.1	5	5.3	7	7.4	4	3
4	0	0	0	0	1	1.0	1	0
Missing	2	1.2	1	1.0	0	0	0	0
Number of lesions (adapted WHO classification) [9]^e^								
0	3	1.8	3	3.2	1	1.0	1	0
1–4	59	34.9	32	33.7	23	24.2	15	8
5–24	89	52.7	47	49.5	47	49.5	23	24
25–100	17	10.1	12	12.6	15	15.8	13	2
> 100	1	0.6	1	1.0	1	1.1	1	0
Missing	0	0	0	0	8	8.4	8	0

ART: antiretroviral therapy; HIV: human immunodeficiency virus; IQR: interquartile range; MSM: men who have sex with men; PrEP: pre-exposure prophylaxis; WHO: World Health Organization.

^a^ All patients living with HIV in this study were on ART.

^b^ Denominators provided indicate all HIV-negative patients in the respective group studied.

^c^ Observed complications: secondary skin infection, tonsillitis, proctitis and urethritis.

^d^ WHO performance status: 0 - Fully active, able to carry on all pre-disease performance without restriction. 1 - Restricted in physically strenuous activity but ambulatory and able to carry out work of a light or sedentary nature, e.g. light house work, office work. 2 - Ambulatory and capable of all self-care but unable to carry out any work activities. Up and about more than 50% of waking hours. 3 - Capable of only limited self-care, confined to bed or chair more than 50% of waking hours. 4 - Completely disabled. Cannot carry on any self-care. Totally confined to bed or chair. 5 - Dead. [17]

^e^ Number of lesions at first visit included visible lesions of any stage (vesicle pustule, ulcer, scab or scar) on the skin and mucosa, and were diagnosed and counted by a physician. Number of lesions at follow-up visit reflected the estimated number of lesions observed during the entire mpox episode, reported by the patient.

Data were collected through standardised questionnaires during study visits; Supplementary Material S3 contains the questionnaires for physicians (part 2) and patients (part 1). Not all patients answered questions regarding fatigue and mental health, as these questions were added after revision of the questionnaire on 9 August 2022. Patients were considered to have medium-term mpox-associated morbidity if they reported persistent anorectal symptoms or sequelae, persistent genital symptoms or sequelae, worsening of fatigue, worsening of mental health problems or loss of physical fitness. These criteria were chosen based on our clinical experience with treating acute mpox patients [8], literature on mpox scar formation in endemic countries [9] and fatigue and loss of physical fitness as possible symptoms after viral infections in general [10–13]. In addition, we explored mental health problems as possible consequences of self-isolation and associated stigmatisation. Descriptive statistical analysis and logistic regression models were performed in Stata (v14.0, StataCorp). 

Overall, 42/61 patients in the early and 22/34 in the late group reported medium-term mpox-associated morbidity (Table 2).

**Table 2:** Health status assessment of mpox patients at follow-up 3–20 weeks after symptom onset, Antwerp, Belgium, June–November 2022 (n = 95)

Health status	Follow-up group (early and late) (n = 95)	3–6 weeks (early) (n = 61)	7–20 weeks (late) (n = 34)
	n	n	n
Follow-up interval after symptom onset, median days (IQR)	32 (26–51)	28 (23–32)	63 (49–91)
Persistent symptoms^a^	64	42	22
**Anorectal symptoms**			
Anorectal symptoms during acute episode	47	33	14
- Proctitis during acute episode (clinical diagnosis)	37	24	13
No anorectal symptoms during acute episode	48	28	20
Ongoing anorectal symptoms among patients with anorectal problems during acute episode (multiple answers possible)	25	17	8
- Peri-anal scars at follow-up	4	1	3
- Anal pain at rest at follow-up	13	10	3 (day^b^ 44, 58, 90)
- Loss of sensitivity due to anal lesions at follow-up	1	0	1 (day^b^ 44)
- Anal pain during defaecation at follow-up	14	12	2 (day^b^ 58, 90)
Anal receptive sex at follow-up	19	11	8
- Anal pain during receptive sex at follow-up	7/19	4/11	3/8 (day^b^ 44, 96, 138)
**Genital symptoms**			
Patients reporting genital lesions during acute episode	45	32	13
- Paraphimosis during acute episode	4	3	1
No genital symptoms during acute episode	50	29	21
Ongoing genital symptoms at follow-up among patients with genital problems during acute episode (multiple answers possible)	18	12	6
- Genital scars at follow-up	11	7	4
- Loss of sensitivity due to genital lesions at follow-up	1	0	1 (day^b^ 79)
- Continued genital pain at rest at follow-up	9	8	1 (day^b^ 107)
Having had erection since acute episode	32	21	11
- Genital pain during erection at follow-up	8/32	7/21	1/11 (day^b^ 132)
Having had penetrative sex since acute episode	20	11	8
- Genital pain during penetrative sex at follow-up	4/20	4/11	0/8
**Physical fitness, fatigue and mental health**			
Loss of physical fitness since acute episode			
- None	46	28	18
- Mild (normal activities possible)	25	17	8
- Moderate (no sport possible)	10	8	2
- Severe (no normal activities possible)	1	1	0
- Missing answer	13	7	6
Worsening or new-onset of fatigue^c^ since acute episode^d^	19/55	14/36	5/19 (day^b^ 43, 58, 71, 131, 132)
Worsening or new-onset of mental problems^e^ since acute episode^d^	11/55	10/36	1/19 (day^b^ 88)

^a^ Symptoms include scars, anorectal and genital symptoms, loss of physical fitness, fatigue and mental health issues.

^b^ Day of follow-up visit after onset of symptoms of the acute episode.

^c^ For symptoms of fatigue, patients were also asked to fill in the Fatigue Severity Scale (FSS). Data not shown.

^d^ Questions about fatigue and mental health problems were introduced after revision of the questionnaire on 9 August 2022 resulting in a lower number of sample size. Patients were asked if they had fatigue or mental health problems before onset of mpox. If they had pre-existing problems, they were asked if symptoms worsened since start of mpox. If they had no pre-exiting problems, they were asked if they had a new onset of problems since mpox. Worsening of existing problems and new onset of problems were then during the analysis combined as problems triggered by mpox. See questionnaires in Supplementary Material S3.

^e^ For symptoms of depression, anxiety and stress, patients were also asked to fill in the Depression Anxiety Stress Scale 21 (DASS-21). Data not shown.

### Post-acute symptoms in the early follow-up group

**Figure 1:**
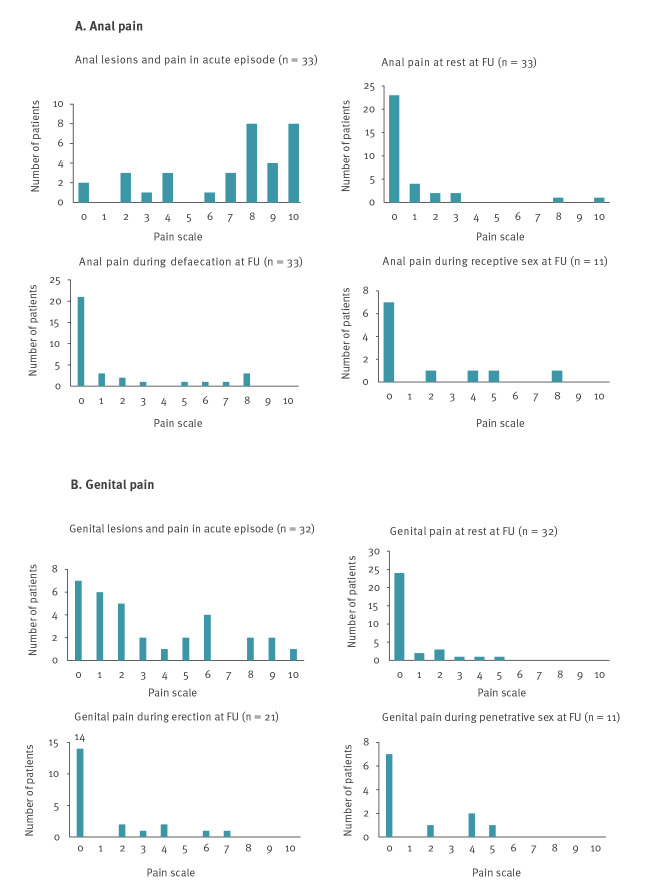
Persistent anal (n = 33) and genital symptoms (n = 32) in mpox patients at follow-up 3–6 weeks after symptom onset, Antwerp, Belgium, June–November 2022

Of the 61 mpox patients in the early group, 33 had (peri)anal lesions and/or proctitis during the acute disease. Among those, 17 had residual anal pain at follow-up: 10/33 at rest and 12/33 during defaecation. Thirty-two of the early group had genital lesions during the acute infection, of whom 12 reported persistent genital signs or symptoms 3–6 weeks later; 7 had genital scars and 8 genital pain at rest. Among 11 patients who had resumed sexual activity, four reported anal pain during receptive anal sex and another four had genital pain during penetrative sex (Figure 1 and Table 2). Nine in the early group reported moderate to severe loss of physical fitness compared with their pre-illness condition (Table 2). Among the 36 patients who were questioned regarding fatigue and mental health, 14 reported worsened or new self-perceived fatigue and 10 worsened or new mental health problems. Three patients had a Depression Anxiety Stress Scale 21 (DASS-21) score compatible with moderate to severe anxiety, three with moderate to severe depression and two with an elevated stress level (data not shown).

FU: follow-up.

Pain is based on a scale of 0–10, where 10 = maximum pain.

### Persistent symptoms in the late follow-up group

**Figure 2:**
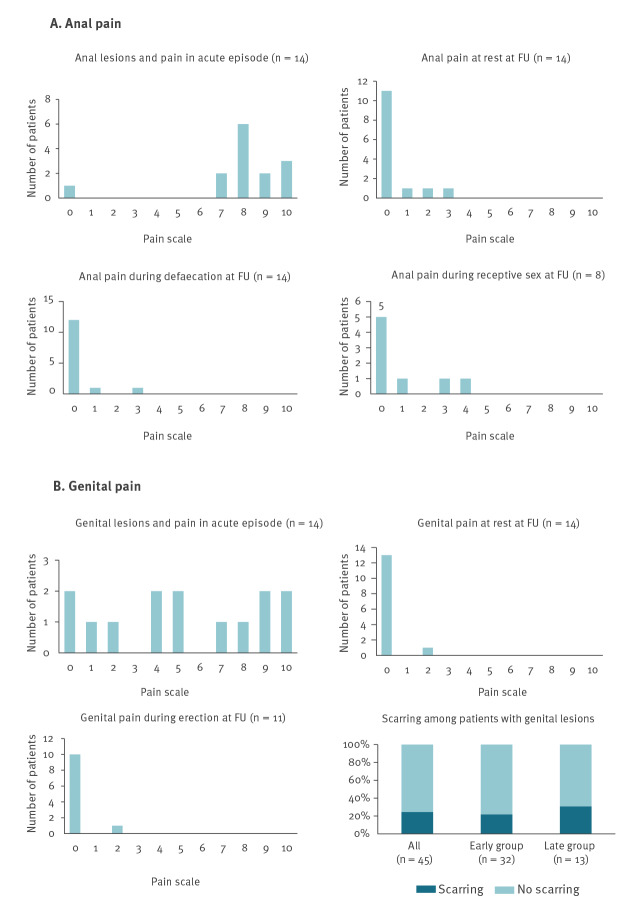
Persistent anal and genital symptoms (n = 14) in mpox patients at follow-up 7–20 weeks after symptom onset, Antwerp, Belgium, June–November 2022

In the late group, 14 patients had (peri)anal lesions and/or proctitis during the acute disease, of which eight reported residual anorectal signs or symptoms: anal pain at rest (n = 3) and/or during defaecation (n = 2), anorectal pain during receptive sex (n = 3), and scarring (n = 3) (Figure 2).

FU: follow-up.

Pain is based on a scale of 0–10, where 10 = maximum pain. Early group: FU visit 3-6 weeks after onset of symptoms; late group: FU visit 7-20 weeks after onset of symptoms.

At diagnosis, 13 late follow-up patients had genital lesions. Six had residual genital problems at follow-up: genital scars (n = 4), loss of penile sensitivity (n = 1), pain during erection (n = 1) and genital pain at rest (n = 1).

Two late follow-up patients reported moderate to severe loss of physical fitness compared with their pre-mpox condition. Among the 19 patients who were questioned regarding fatigue and mental health, five reported worsening or new onset of fatigue and one reported a worsened or new mental health issue (i.e. mild anxiety). Supplementary Table S1 shows all patients with ongoing symptoms presenting after 6 weeks. Although no clear pattern emerged, persistent pain, especially in the anorectal region, seemed associated with severe pain during the acute episode. Nevertheless, severe pain in the beginning did not always result in persistent symptoms.

### Risk factors for medium-term mpox-associated morbidity

Among all 95 participants of our study, the presence of medium-term mpox-associated morbidity was negatively associated with age (odds ratio (OR): 0.95, 95% confidence interval (CI): 0.91–0.99, p = 0.029). We assumed confounding by vaccine status and split age into two groups (< 47 years and ≤ 47 years). No association with age was found in the stratified groups but the younger age group showed four times higher risk for mpox-associated morbidity compared with the older group (OR: 4.41, 95% CI: 1.62–12.03, p = 0.004). Belonging to the younger group was also associated with a 10-times higher risk to develop fatigue (n = 55, OR: 10.4, 95% CI: 1.20–90.09, p = 0.034).

HIV status was not associated with mpox-associated morbidity, nor were the presence of systemic symptoms or complications the number of lesions or WHO performance status during the acute illness. See Supplementary Table S2 for a risk factor and log regression analysis in patients with any persistent symptom and the variables mentioned above. However, genital scarring seemed to be a risk factor for persistent genital pain at follow-up (n = 45, OR: 6.46, 95% CI: 1.33–31.32, p = 0.021).

## Discussion

Two thirds of former mpox patients in our study presented with medium-term mpox-associated morbidity 3 to 20 weeks after their initial infection. Half of those who had anorectal pain at diagnosis still had anorectal complaints at follow-up and about one third of those with prior genital lesions had persistent genital issues at follow-up. Other common morbidities included loss of physical fitness and fatigue since the acute mpox episode, which were reported in the early as well as in the late follow-up group of patients. The latter is not surprising, as post-infectious fatigue is a common long-term consequence of many other infectious diseases such as coronavirus disease (COVID-19) or dengue [10-13]. Post-infectious fatigue in COVID-19 and dengue was associated with disease severity [13,14]. In our cohort, variation of mpox disease severity was very limited. However, older age was associated with a lower probability of medium-term mpox-associated morbidity and fatigue. Although the sample size was small and the older age group underrepresented, this observation may be a consequence of smallpox vaccination in individuals born in Belgium before 1976. It is unclear to what extent prior smallpox vaccination has mitigated the impact of mpox, as information about the childhood vaccination status of our participants was inconsistent and unreliable. A possible protective effect of smallpox vaccination against long-term mpox symptoms has not been described but a possible association of a milder course of mpox disease and history of a previous smallpox vaccination has been discussed by some authors [15,16].

New-onset or worsening of anxiety, stress and/or depressive symptoms were found in 20% of participants, mainly in the early follow-up group. Different factors might influence anxiety, stress and depressive mood after mpox; patients were confronted with a new and stigmatising disease, no definitive treatment was available and patients had to self-isolate for 3 weeks.

A major limitation of this study is that 44% of the mpox patients did not return for follow-up. Resolution of symptoms may have been an important reason for not returning to the clinic. Hence, patients with persisting symptoms may be overrepresented in our study. Furthermore, coherent follow-up of all patients during the mpox epidemic was difficult, and several patients presented later than planned. This resulted in a smaller sample size and a larger variation in time to follow-up than foreseen. Nevertheless, to the best of our knowledge there are no comparable prospective data published on the convalescence phase after acute Clade IIb mpox disease. 

## Conclusion

A high proportion of mpox patients presented with medium-term mpox-associated morbidity. Although most described health problems might eventually resolve, persistent symptoms may result in a significant reduction in quality of life and require further investigation in longitudinal studies. Most importantly, clinicians should be aware of the pain, scarring and mental health issues that may persist after a seemingly self-limiting illness like Clade IIb mpox. 

## Supplementary Data

363000 Supplement
