# Supplementary material for: Persistent morbidity in Clade IIb mpox patients: interim results of a long-term follow-up study, Belgium, June to November 2022
Source: Euro Surveill. 2023 Feb 16;28(7):2300072. doi: 10.2807/1560-7917.ES.2023.28.7.2300072 (PMC9936596; doi:10.2807/1560-7917.ES.2023.28.7.2300072)
Supplement: Supplement [file 23-00072_BERENS-RIHA_SUPPLEMENT.pdf]

"This supplementary material is hosted by *Eurosurveillance* as supporting information alongside the article "Persistent morbidity in Clade IIb mpox patients: interim results of a long-term follow-up study, Belgium, June to November 2022", on behalf of the authors, who remain responsible for the accuracy and appropriateness of the content. The same standards for ethics, copyright, attributions and permissions as for the article apply. Supplements are not edited by *Eurosurveillance* and the journal is not responsible for the maintenance of any links or email addresses provided therein."

Supplementary Table S1. Case series with patients having persistent symptoms > 6 weeks (43-164 days) after onset of symptoms

Patient	FU-interval (days)[1]	Living with HIV	Other STI Co-Infections	Number of lesions[2]	Age (years)	Systemic symptoms[3]	Complications[4]	Acute anal pain during mpox	Any persistent anal problems	Acute genital pain during mpox	Any persistent genital problems	Persistent fatigue	Persistent loss of physical fitness	Persistent mental health problems
**1**	43	Yes	HIV (new)	2	30-40	Yes	No	No	N/A[5]	No	N/A	**Yes**	**Yes (mild)**	No
**2**	44	No	Lues	2	50-60	Yes	No	8/10[7]	**Pain at rest, sensitivity loss**	1/10	No	No	No	No
**3**	44	No	No	2	50-60	No	No	No	N/A	No	N/A	No	**Yes (mild)**	No
**4**	44	No	No	2	30-40	Yes	Proctitis[6]	8/10	**Pain during anal sex**	No	N/A	No	No	No
**5**	49	No	No	2	20-30	Yes	No	No	N/A	No	N/A	No	**Yes (moderate)**	No
**6**	52	No	No	2	30-40	Yes	No	No	N/A	9/10	No	No	**Yes (mild)**	No
**7**	56	Yes	No	1	50-60	Yes	No	No	N/A	No	N/A	No	**Yes (mild)**	No
**8**	58	No	No	2	40-50	Yes	Proctitis	9/10	**Pain at rest**	No	N/A	**Yes**	**Yes (moderate)**	No
**9**	60	Yes	No	2	30-40	Yes	No	No	N/A	9/10	**Scar**	No	No	No
**10**	65	No	No	1	20-30	Yes	Bacterial superinfection	No	N/A	No	N/A	No	**Yes (mild)**	No
**11**	71	No	No	2	30-40	Yes	Proctitis, Tonsilitis,	7/10	No	No	N/A	**Yes**	No	No

							Urinary retention							
**12**	73	No	No	2	40-50	Yes	Proctitis	9/10	No	No	N/A	No	**Yes (mild)**	No
**13**	79	Yes	No	2	60-70	Yes	Urethritis	No	N/A	4/10	**Sensitivity loss**	No	**Yes (mild)**	No
**14**	88	No	No	1	20-30	Yes	No	No	N/A	10/10	No	No	No	**Yes (mild anxiety)**
**15**	90	Yes	No	2	40-50	Yes	Proctitis	8/10	**Pain at rest/during defaecation**	No	N/A[5]	No	No	No
**16**	93	Yes	Lues	2	30-40	Yes	Proctitis	10/10	**Scars**	No	N/A	no data	No	No
**17**	96	No	No	2	30-40	Yes	No	8/10	**Pain during anal sex**	No	N/A	No	No	No
**18**	99	Yes	No	1	50-60	Yes	No	No	N/A	7/10	**Scar**	No	No	No
**19**	107	No	No	2	30-40	No	No	No	N/A	10/10	**Scar, pain at rest**	No	No	No
**20**	131	No	No	2	20-30	Yes	Proctitis	10/10	**Scars**	No	N/A	**Yes**	**Yes (mild)**	No
**21**	132	No	No	3	30-40	Yes	No	No	N/A	4/10	**Scar, pain during erection**	**Yes**	No	No
**22**	138	Yes	No	3	40-50	Yes	Proctitis	10/10	**Scars**	No	N/A	No	No	No
**23**	142	No	No	1	30-40	Yes	Proctitis	8/10	No	No	N/A	No	**Yes (mild)**	No
**24**	146	No	No	3	40-50	Yes	Proctitis	10/10	**Pain during anal sex**	No	N/A	No	No	No
**25**	164	No	No	2	40-50	Yes	No	No	N/A	No	N/A	**Yes**	**Yes (moderate)**	**Yes (mild stress)**

[1]Patients 23-25 are not included in the main analysis of 95 patients. Cut-off was 20 weeks. [2]Number of lesions: 0=none, 1=1-4, 2=5-24, 3=25-100, 4>100. [3]Systemic symptoms: fever, chills, fatigue, headache, muscle pain, joint pain, systemic lymphadenopathy. [4]Complications: Secondary skin infection, Tonsillitis, Pneumonia, Proctitis, Urethritis, Urinary retention, Paraphimosis. [5]N/A: not applicable (patient never had any anal or genital lesions). [6]Clinical diagnosis. [7]Maximal pain on a scale of 10 during the acute mpox episode

Note: Loss of physical fitness, fatigue (Fatigue Severity Scale (FSS)) and mental health problems (Depression Anxiety Stress Scale 21 items (DASS21)) are very subjective symptoms and only with great caution attributable to the acute mpox disease. Only new onset or worsening of symptoms was reported but other causes during the follow-up are possible. For example fatigue due to an unrecognized Covid-19 episode.

Supplementary Table S2. Risk factor analysis for the outcome "any persistent symptom" (n=95)

**Any persistent symptoms**	**n/N (%)**	**OR (95% CI)[1]**	**p-value**
Born 1975 or earlier	9/22 (40.9)		**0.004**
Born after 1975	55/73 (75.3)	4.41 (1.62-12.03)	
HIV negative	47/67 (70.2)		0.373
HIV positive	17/28 (60.71)	0.66 (0.26-1.65)	
Number of lesions			
0	1/1 (100.0)		
1-4	14/23 (60.9)	0.39 (0.09-1.77)	0.222
5-25	33/47 (70.2)	0.59 (0.14-2.42)	0.463
26-100	12/15 (80.0)	1	n/a
>100	1/1 (100.0)	1	n/a
No complications during acute mpox	27/38 (71.1)		0.532
Any complications during acute mpox	37/57 (64.9)	0.75 (0.31-1.83)	
No proctitis during acute mpox	37/58 (63.8)		0.354
Proctitis during acute mpox	27/37 (73.0)	1.53 (0.62-3.76)	
No systemic mpox symptoms	11/14 (78.6)		0.516
Systemic mpox symptoms	44/63 (69.8)	0.63 (0.16-2.52)	
WHO performance status			
0=Fully active	19/31 (61.3)		
1=Restricted in physically strenuous activity, capable of house work, office work	25/33 (75.8)	1.97 (0.67-5.78)	0.215
2=Ambulatory and capable of all selfcare but no work activities	14/23 (60.9)	0.98 (0.32-2.97)	0.975
3=Capable of only limited selfcare, confined to bed/chair > 50% of waking hours	5/7 (71.4)	1.58 (0.26-9.47)	0.617
4=Completely disabled. Cannot carry on any selfcare. Totally confined to bed/chair	1/1 (100.0)	1	n/a
5=Dead	0	n/a	

[1] Logistic regression

Supplementary material S3. Questionnaires used in the mpox follow-up study for the investigator (part 2. physician) and the patient (part 1. patient)

MPX Follow Up

Admin no _ _ _ _

Part 2. PHYSICIAN

Date of visit: ___/___/___

A. Participant in ASSESS ☐ No ☐ Yes → ID ASSESS __ __ __ __

B. Complaints

1. Experiencing symptoms due to positive PCR for MPXV?

☐ No ☐ Yes → last day of complaints: __ __ /__ / 2022

2. Admission to hospital?

☐ No ☐ Yes → request medical report

3. General symptoms during infection?

☐ No ☐ Yes → duration of symptoms: ______ days

4. Maximum debilitation according to the WHO scale (during illness):

☐ 0 = fully active, able to perform all pre-disease performance without restriction

☐ 1 = limited in physically demanding activities, but ambulatory and able to carry out light work

☐ 2 = ambulatory and capable of all self-care but unable to carry out any work activities

☐ 3 = only capable of limited self-care, > 50% of waking hours confined to bed or chair

☐ 4 = completely disabled. Cannot carry out any self-care. Completely confined to bed or chair.

5. Complications?

☐ No
☐ Yes:
- ☐ Secondary bacterial infection (superficial/deep tissue):
  - ☐ skin (face) ☐ skin (other than face)
  - ☐ anal ☐ other: _______________
- ☐ Abscess (with need for operative opening), localization:______________________________
- ☐ Proctitis
- ☐ Urethritis
- ☐ Pneumonia
- ☐ Tonsillitis
- ☐ Paraphimosis
- ☐ Urinary retention
- ☐ Conjuctivitis
- ☐ Other:____

6. Other STI diagnosed as co-infection?

☐ No
☐ Yes:
- ☐ Syphilis (new) ☐ Gonorrhea
- ☐ HSV ☐ Chlamydia
  - ☐Non-LGV ☐LGV
- ☐ HIV (new) ☐ Hepatitis B (new)
- ☐ Hepatitis C (new)

7. Antibiotics given?

☐ No
☐ Yes:
Name: ☐ Ceftriaxone ☐ Doxycycline ☐ Penicillin ☐ Azithromycin
☐ Other:___________________________________

Indication: ☐ Bacterial superinfection of the skin (without culture/antibiogram)
☐ Proctitis ☐Urethritis ☐ Genital ulcer ☐Other:_________________

8. Anti-inflammatory treatment for proctitis?

☐ No ☐ Yes
☐Mesalazine(Colitofalk/Pentasa) rectal suspension
☐Other:____

9. MPX treatment given?

☐ No ☐ Yes → date:____/____/2022
→ which one?
☐ Vaccination
☐ Tecovirimat 14 days ☐ Tecovirimat other schedule
☐ Other treatment:_______________________________________

10. MPX PEP given?

☐ No ☐ Yes → date:____/____/2022
→ which one?
☐ Vaccination
☐ Tecovirimat 14 days ☐Tecovirimat other schedule
☐ Other treatment:_______________________________________

11. MPX PrEP given?

☐ No ☐ Yes → date:____/____/2022

→ which one?

☐Imvanex ☐Jynneos ☐Other:______________________

12. HIV treatment, PrEP, or other antivirals taken before the onset of symptoms?

☐No ☐ Yes: ____

13. Needed surgery due to scarification?

☐ No ☐ Yes → request medical report

____

Date

____

Name of physician Signature of physician

Part 1. PATIENT

A. Anal lesions

1. Did you experience anal discomfort or pain during the monkeypox infection?

☐ No ☐ Yes ☐ Not sure

2. At this moment, are there any strictures or scarifications of the anus or rectum that were not present before the monkeypox infection?

☐ No ☐ Yes ☐ Not sure

3. At this moment, are you experiencing any anal discomfort or pain that was not present before the monkeypox infection?

☐ No ☐ Yes ☐ Not sure

4. What is the highest level of anal pain you've experienced during the monkeypox infection?

(0 = no pain; 10 = worst pain ever)

☐0 ☐1 ☐2 ☐3 ☐4 ☐5 ☐6 ☐7 ☐8 ☐9 ☐10

5. What is the highest level of anal pain you've experienced in the past 24h?

(0 = no pain; 10 = worst pain ever)

☐0 ☐1 ☐2 ☐3 ☐4 ☐5 ☐6 ☐7 ☐8 ☐9 ☐10

6. What is the level of anal pain you've experienced during your last defecation?

(0 = no pain; 10 = worst pain ever)

☐0 ☐1 ☐2 ☐3 ☐4 ☐5 ☐6 ☐7 ☐8 ☐9 ☐10

7. What is the level of pain during your last receptive anal sexual contact (= bottom contact) since the onset of the infection? (0 = no pain; 10 = worst pain ever)

☐I didn't have receptive anal sexual contact since the onset of the monkeypox infection

☐0 ☐1 ☐2 ☐3 ☐4 ☐5 ☐6 ☐7 ☐8 ☐9 ☐10

8. At this moment, do you experience a loss of sensation in the anal region that was not present before the monkeypox infection?

☐ No

- ☐ Yes:
  - ☐ a little numbness
  - ☐ a significant numbness
  - ☐ a complete numbness

B. Continence problems (SMIS scale)

	never	rarely	sometimes	weekly	daily
Do/did you experience problems holding solid stools…					
before the monkeypox infection?	☐0	☐1	☐2	☐3	☐4
during the monkeypox infection?	☐0	☐1	☐2	☐3	☐4
after the monkeypox infection?	☐0	☐1	☐2	☐3	☐4
Do/did you experience problems holding loose stools…					
before the monkeypox infection?	☐0	☐1	☐2	☐3	☐4
during the monkeypox infection?	☐0	☐1	☐2	☐3	☐4
after the monkeypox infection?	☐0	☐1	☐2	☐3	☐4
Do/did you experience problems holding bowel gas…					
before the monkeypox infection?	☐0	☐1	☐2	☐3	☐4
during the monkeypox infection?	☐0	☐1	☐2	☐3	☐4
after the monkeypox infection?	☐0	☐1	☐2	☐3	☐4
Did these continence problems lead to a change in lifestyle…					
before the monkeypox infection?	☐0	☐1	☐2	☐3	☐4
during the monkeypox infection?	☐0	☐1	☐2	☐3	☐4
after the monkeypox infection?	☐0	☐1	☐2	☐3	☐4

	No	Yes
Do/did you need to wear incontinence material (e.g., fecal absorbent pad)…		
before the monkeypox infection?	☐0	☐2
during the monkeypox infection?	☐0	☐2
after the monkeypox infection?	☐0	☐2
Do/did you need medication for constipation…		
before the monkeypox infection?	☐0	☐2
during the monkeypox infection?	☐0	☐2
after the monkeypox infection?	☐0	☐2
Do/did you experience an inability in delaying defecation…		
before the monkeypox infection?	☐0	☐2
during the monkeypox infection?	☐0	☐2
after the monkeypox infection?	☐0	☐2

C. Genital lesions

1. Did you experience any discomfort or pain at the penis and/or scrotum during the monkeypox infection?

☐ No ☐ Yes ☐ Not sure

2. At this moment, are there adhesions or scars on the penis and/or foreskin that were not present before the monkeypox infection?

☐ No ☐ Yes ☐ Not sure

3. At this moment, are you experiencing any discomfort or pain of the penis and/or scrotum that was not present before the monkeypox infection?

☐ No ☐ Yes ☐ Not sure

4. What is the highest level of pain at the penis and/or scrotum that you experienced during the infection?

(0 = no pain; 10 = worst pain ever)

☐0 ☐1 ☐2 ☐3 ☐4 ☐5 ☐6 ☐7 ☐8 ☐9 ☐10

5. What is the highest level of pain at the penis and/or scrotum that you experienced in the past 24h?

(0 = no pain; 10 = worst pain ever)

☐0 ☐1 ☐2 ☐3 ☐4 ☐5 ☐6 ☐7 ☐8 ☐9 ☐10

6. What is the level of pain at penis and/or scrotum that you experienced during your last erection since the start of the monkeypox infection? (0 = no pain; 10 = worst pain ever)

☐ I didn't have an erection since the beginning of the infection

☐0 ☐1 ☐2 ☐3 ☐4 ☐5 ☐6 ☐7 ☐8 ☐9 ☐10

7. What is the level of pain at the penis and/or scrotum during your last insertive sexual contact (= top contact) since the start of the monkeypox infection? (0= no pain;10= worst pain ever)

☐ I didn't have an insertive sexual contact since the start of the monkeypox infection

☐0 ☐1 ☐2 ☐3 ☐4 ☐5 ☐6 ☐7 ☐8 ☐9 ☐10

8. Are you currently experiencing a loss of sensation at the penis and/or scrotum that was not present before the monkeypox infection?

- ☐ No
- ☐ Yes:
  - ☐ little numbness
  - ☐ significant numbness
  - ☐ complete numbness

D. Fatigue (FSS scale)

1. Did you experience symptoms of fatigue before the monkeypox infection?

☐ No ☐ Yes ☐ Not sure

2. Have you experienced new or increased symptoms of fatigue since the monkeypox infection?

☐ No ☐ Yes ☐ Not sure

3. To what extent do the following statements apply to you since the onset of the monkeypox infection?

(1 = I totally disagree; 4 = I'm neutral; 7 = I totally agree)

My motivation is lower when I am fatigued	☐1	☐2	☐3	☐4	☐5	☐6	☐7
Exercise brings on my fatigue	☐1	☐2	☐3	☐4	☐5	☐6	☐7
I am easily fatigued	☐1	☐2	☐3	☐4	☐5	☐6	☐7
Fatigue interferes with my physical functioning	☐1	☐2	☐3	☐4	☐5	☐6	☐7
Fatigue causes frequent problems for me	☐1	☐2	☐3	☐4	☐5	☐6	☐7
My fatigue prevents sustained physical functioning	☐1	☐2	☐3	☐4	☐5	☐6	☐7
Fatigue interferes with carrying out certain duties and responsibilities	☐1	☐2	☐3	☐4	☐5	☐6	☐7
Fatigue is amongst my three most disabling symptoms	☐1	☐2	☐3	☐4	☐5	☐6	☐7
Fatigue interferes with my work, family or social life	☐1	☐2	☐3	☐4	☐5	☐6	☐7

E. Mental health ($DASS_{21}$ scale)

1. Did you experience any mental problems before infection with the monkeypox virus?

☐ No ☐ Yes:

- ☐ Fear
- ☐ Depressive feelings
- ☐ Mood swings
- ☐ Dependence on drugs or alcohol
- ☐ Behavioral problems (e.g., anger control problems)
- ☐ Eating disorders (e.g., overeating or anorexia)
- ☐ Concentration disorders, abnormal forgetfulness

2. If yes: Have these symptoms increased since the monkeypox infection?

☐ No ☐ Yes

3. Do you experience mental problems since the monkeypox infection?

☐ No ☐ Yes:

- ☐ Fear
- ☐ Depressive feelings
- ☐ Mood swings
- ☐ Dependence on drugs or alcohol
- ☐ Behavioral problems (e.g., anger control problems)
- ☐ Eating disorders (e.g., overeating or anorexia)
- ☐ Concentration disorders, abnormal forgetfulness

4. To what extent do the following statements apply to you since the onset of the monkeypox infection?

0 = Does not apply to me at all
1 = Applies to me to some degree, or some of the time
2 = Applies to me to a considerable degree, or a good part of the time
3 = Applies to me very much, or most of the time

1. I find it hard to calm myself down	☐0	☐1	☐2	☐3
2. I notice dryness of my mouth	☐0	☐1	☐2	☐3
3. I don’t seem to experience any positive feeling at all	☐0	☐1	☐2	☐3
4. I experience breathing difficulty (e.g., excessively rapid breathing, breathlessness in the absence of physical exertion)	☐0	☐1	☐2	☐3
5. I find it difficult to take the initiative to do things	☐0	☐1	☐2	☐3
6. I tend to overreact to situations	☐0	☐1	☐2	☐3
7. I experience trembling (e.g. of the hands)	☐0	☐1	☐2	☐3
8. I feel that I am using a lot of nervous energy	☐0	☐1	☐2	☐3
9. I worry about situations in which I will panic and make a fool of myself	☐0	☐1	☐2	☐3
10. I feel like I have nothing to look forward to	☐0	☐1	☐2	☐3
11. I notice that I am very restless and/or agitated	☐0	☐1	☐2	☐3
12. I find it difficult to relax	☐0	☐1	☐2	☐3
13. I feel down-hearted and blue	☐0	☐1	☐2	☐3
14. I am intolerant of anything that keeps me from getting on with what I am doing	☐0	☐1	☐2	☐3
15. I feel I'm close to panick	☐0	☐1	☐2	☐3
16. I am unable to become enthusiastic about anything	☐0	☐1	☐2	☐3
17. I feel I am not worth much as a person	☐0	☐1	☐2	☐3
18. I feel that I am rather touchy	☐0	☐1	☐2	☐3
19. I am aware of my heart rate in the absence of physical activity (e.g., sense of heart rate increase or heart missing a beat)	☐0	☐1	☐2	☐3
20. I feel scared without any good reason	☐0	☐1	☐2	☐3
21. I feel like my life is meaningless	☐0	☐1	☐2	☐3

F. Physical Fitness.

1. Are you experiencing a loss in physical fitness since the monkeypox infection?

☐ No ☐ Moderate (no sport possible)

☐ Mild (not restricting normal life) ☐ Severe (no normal activities possible)

Thank you very much for filling in this questionnaire! This information is very important to us.

Are you interested in receiving this questionnaire monthly? By doing so, we will collect useful information to increase our knowledge of the disease and optimize long-term care.

If agreed, please enter your mail address here: -

____
